# How does oestradiol influence the AVT/IT system in female round gobies during different reproductive phases?

**DOI:** 10.1242/bio.024844

**Published:** 2017-08-31

**Authors:** Hanna Kalamarz-Kubiak, Magdalena Gozdowska, Tatiana Guellard, Ewa Kulczykowska

**Affiliations:** Department of Genetics and Marine Biotechnology, Institute of Oceanology Polish, Academy of Sciences, Powstańców Warszawy 55, 81-712 Sopot, Poland

**Keywords:** Oestradiol, AVT, IT, *In vitro* gradient perfusion, Genomic and non-genomic pathways, Female round goby

## Abstract

In this *in vitro* gradient perfusion study, we determined whether there is a functional relationship between oestradiol and the arginine vasotocin/isotocin (AVT/IT) system in the female round goby (*Neogobius melanostomus*). Brain explants were perfused in medium supplemented with 17β-oestradiol (E_2_) at doses mimicking the plasma levels of this hormone in nature during the spawning-capable phase and regressing phase. We aimed to establish which pathway, genomic or non-genomic, is involved in this mechanism in different reproductive phases. For this purpose, brain explants were perfused in medium supplemented with Fulvestrant (ICI 182.780) or Actinomycin D (Act D) separately or in combination with E_2_. The contents of AVT and IT in the perfusion media were determined using high-performance liquid chromatography (HPLC) with fluorescence and UV detection. During the spawning-capable phase, the effect of E_2_ on AVT release is mediated through oestrogen receptors (ERs) via both genomic and non-genomic pathways, while IT release is mediated through ERs via a genomic pathway only. In the regressing phase, release of both nonapeptides is mediated through ERs via a genomic pathway. This is the first study to present a feasible mechanism of oestradiol action on the AVT/IT system in female fish during different phases of the reproductive cycle.

## Abstract (precis)

**Summary:** This *in vitro* gradient perfusion study presents a presumable mechanism of 17β-oestradiol action on the AVT/IT system in the female round goby during different phases of the reproductive cycle.

## INTRODUCTION

Oestradiol, which is synthesized in gonads and in the brain, is crucial to the neuroendocrine control of reproduction and sexual behaviour in vertebrates (Knobil and Neill, 1994; Compagnone and Mellon, 2000). The functions of oestrogens include the regulation of oocyte growth within the gonads, secondary sex characteristics and behaviour (Nagahama et al., 1995). Oestradiol acts at the level of the brain and pituitary, and influences the secretion of gonadotropin-releasing hormone and other neuropeptides that control reproduction and reproductive behaviour (McEwen et al., 1997; Shupnik, 2002). The fish neuropeptides arginine vasotocin (AVT) and isotocin (IT), like their mammalian homologues arginine vasopressin (AVP) and oxytocin (OT), are also involved in the regulation of reproduction and social behaviour (Moore, 1992; Goodson and Bass, 2001). In fish, the AVT/IT system consists of separate parvo- and magnocellular neurons of the preoptic area (POA), which project fibres to multiple brain areas, such as the ventral telencephalon, diencephalon and various mesencephalic structures, and to the neurohypophysis (Holmqvist and Ekström, 1995; Saito et al., 2004). In Teleostei, AVT has been reported to influence sexual behaviour, including courtship (Salek et al., 2002; Grober and Bass, 2002), aggression (Semsar et al., 2001; Greenwood et al., 2008; Kleszczyńska et al., 2012), vocalization (Goodson and Bass, 2000a) and spawning reflex (Knapp et al., 1999). Less is known about the role of IT in the regulation of sexual behaviour in fish. The investigations on this subject are limited to the species plainfin midshipman fish (*Porichthys notatus*) (Goodson and Bass, 2000b), *Lythrypnus dalli* (Black et al., 2004) and three-spined stickleback (*Gasterosteus aculeatus*) (Kleszczyńska et al., 2012; Kulczykowska and Kleszczyńska, 2014). It has been established that IT is involved in controlling aggression, courtship, final oocyte maturation and/or egg deposition in the three-spined stickleback (Kleszczyńska et al., 2012; Kulczykowska and Kleszczyńska, 2014), and vocal-motor responses in plainfin midshipman fish (Goodson and Bass, 2000b). Because AVT and IT immunoreactivity (-IR) was found within multiple components of the ascending auditory pathway, the modulatory role of both neuropeptides was suggested in fish vocalization (Goodson et al., 2003). However, in female plainfin midshipman fish, only local administration of IT inhibited vocal-motor responses elicited by anterior hypothalamic stimulation, and this effect is reversed by an OT antagonist. In tetrapods and fish, there are indications of possible interactions between oestrogens and AVP/AVT-ergic or OT/IT-ergic systems.

However, studies have focused on the location of these hormones or their receptors in the brain (Axelson and Leeuwen, 1990; Shughrue et al., 1996, 1997; Simonian and Herbison, 1997; Harbovszky et al., 1998; Alves et al., 1998; Foidart et al., 1999). As was mentioned earlier, the teleost brain is well known as the site of *de novo* synthesis of oestrogens, catalysed by a brain-specific aromatase enzyme.

However, to the authors’ knowledge, only Lorenzi et al. (2012) has presented 17β-oestradiol (E_2_) concentration in the brain of *Lythrypnus dalli*. In female *L. dalli*, the gonadal E_2_ concentration was ∼10 times higher than that in the brain. In the brain, there are three oestrogen receptor (ER) subtypes (ER-α, -β1, -β2) recognized in fish. ER-α protein and ER-α mRNA have been localized in the forebrain of rainbow trout (*Oncorhynchus mykiss*) (Kah et al., 1997; Menuet et al., 2003). In Atlantic croaker (*Micropogonias undulates*), ER-α, -β1, -β2 mRNA expression has been found in the suprachiasmatic nucleus of the POA (Hawkins et al., 2000). In addition, the three subtypes of ERs are distributed in the anterior POA, the ventral hypothalamus and the posterior tuberculum of the zebrafish (*Danio rerio*) (Menuet et al., 2002). More information is currently available about the distribution of ERs in the plainfin midshipman fish (*Porichthys notatus*) brain. The anatomical localisation of ER-α was noticed in the telencephalon, POA, diencephalon, anterior hypothalamus and hindbrain; ER-β1 in the telencephalon, POA and hindbrain; ER-β2 in the telencephalon, POA, anterior hypothalamus, midbrain and hindbrain (Forlano et al., 2005; Fergus and Bass, 2013). In fish, ERs are located in brain regions of AVT and IT synthesis, similar to tetrapods, where ERs are located in AVP- and OT-synthesizing cells (Anglade et al., 1994; Holmqvist and Ekström, 1995; Hawkins et al., 2005). Moreover, ERs are distributed in the hypothalamic and extrahypothalamic areas related to the control of reproduction (e.g. mediobasal hypothalamus, ventral telencephalon), where both nonapeptides give projections (Anglade et al., 1994; Goodson and Bass, 2001; Goodson et al., 2003; Forlano et al., 2005; Thompson and Walton, 2009; Fergus and Bass, 2013).

According to current knowledge, oestradiol controls gene expression via ERs by activation of both genomic (nuclear) and non-genomic (extranuclear) pathways. Genomic pathways encompass the classical interactions between ligand-bound ER dimers and oestrogen-responsive elements in target gene promoters (Gronemeyer et al., 2004) and the non-classical pathway, where a ligand activates ER/specificity protein and ER/activating protein-1 complexes (Safe and Kim, 2008). The non-genomic effect is mediated through the membrane ER (mER) ‘family’, consisting of classical ER-α and ER-β or splice variants, ER-X, two G-protein-coupled oestrogen receptors, GPER (also known as GP30) and Gq-mER (Toran-Allerand et al., 2002; Qiu et al., 2003; Micevych and Kelly, 2012). Oestrogens may also exert non-genomic action without receptor involvement through a physicochemical interaction with plasma membrane at only micromolar concentrations (Falkenstein et al., 2000; Simoncini and Genazzani, 2003).

In fish, there is little information on the effects of oestradiol on the synthesis and release of AVT and IT. Available information is related only to seasonal changes in gene expression and immunoreactivity of the nonapeptides, which are probably linked with changes in steroid hormones. In the female grass puffer (*Takifugu niphobles*), brain AVT mRNA expression is augmented during the spawning period (Motohashi et al., 2008). Ota et al. (1999) have demonstrated that increases in AVT mRNA level and AVT-IR in the POA are accompanied by elevation of plasma oestradiol levels in immature female masu salmon (*Oncorhynchus masou*) in November. In female medaka (*Oryzias latipes*) and halfspotted goby (*Asterropteryx semipunctata*), AVT-IR and IT-IR signals in the preoptic–hypothalamic regions in the pre-spawning and spawning phases were stronger than those in the post-spawning phase (Ohya and Hayashi, 2006; Maruska et al., 2007). Based on our studies with sticklebacks (Gozdowska et al., 2006; Kleszczyńska et al., 2012; Kulczykowska and Kleszczyńska, 2014) and round goby (Sokołowska et al., 2015), we presume that there is a functional link between oestradiol and the AVT/IT system in fish during different phases of the reproductive cycle. In a socially controlled situation such as the masculinization process of females, a link between sex steroids and brain AVT and IT was shown in black molly (*Poecilia sphenops*) (Kulczykowska et al., 2015). So far, to the authors' knowledge, there is no evidence that a functional relationship between oestradiol and the AVT/IT system exists in fish. In this study, we determine whether there is a functional relationship between circulating oestradiol and AVT and IT in the female round goby (*Neogobius melanostomus*). We try to establish which pathway, genomic or non-genomic, is involved in this mechanism in different reproductive phases. The *in vitro* gradient perfusion technique is the method of choice (Kalamarz-Kubiak et al., 2011) because it monitors the dynamic hormone secretion and registers even small and shortened fluctuations in hormone secretion before and after treatment. The brain explants are perfused in medium supplemented with E_2_ at doses mimicking the plasma levels of this hormone in nature during different reproductive phases. In the perfusion of brain explants, we use E_2_ separately or in combination with Fulvestrant (ICI 182.780) or Actinomycin D (Act D). Fulvestrant is an ER antagonist (Robertson, 2001), which affects the reproduction processes in fish females (vitellogenesis, oocyte maturation) and males (steroidogenesis in testes) (Bouma et al., 2003; Pang and Thomas, 2009; Nagler et al., 2010). Actinomycin D is an effective inhibitor of ER processing because it directly blocks ER access to a specific region of DNA (Horwitz and McGuire, 1978).

## RESULTS

### Base levels of AVT and IT release into the perfusion media (control) and 17β-oestradiol in plasma during the spawning-capable phase and the regressing phase

Levels of AVT and IT release were significantly higher (*P*<0.001) during the spawning-capable phase than in the regressing phase (Table 1). Plasma E_2_ was significantly higher (*P*<0.001) during oocyte maturation in the spawning-capable phase than during the regressing phase (Table 1).

**Table 1.**
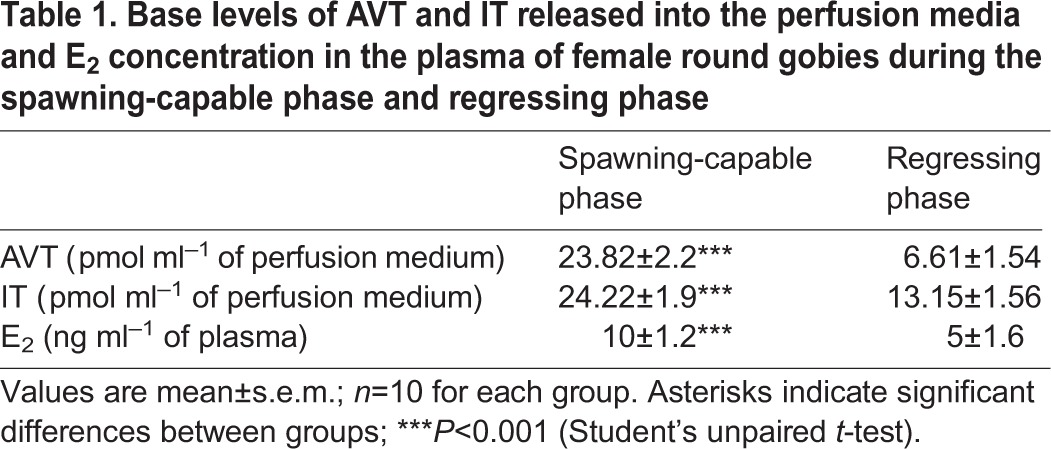
**Base levels of AVT and IT released into the perfusion media and E_2_ concentration in the plasma of female round gobies during the spawning-capable phase and regressing phase**

### The influence of 17β-oestradiol on AVT and IT during the spawning-capable phase

**Fig. 1.**
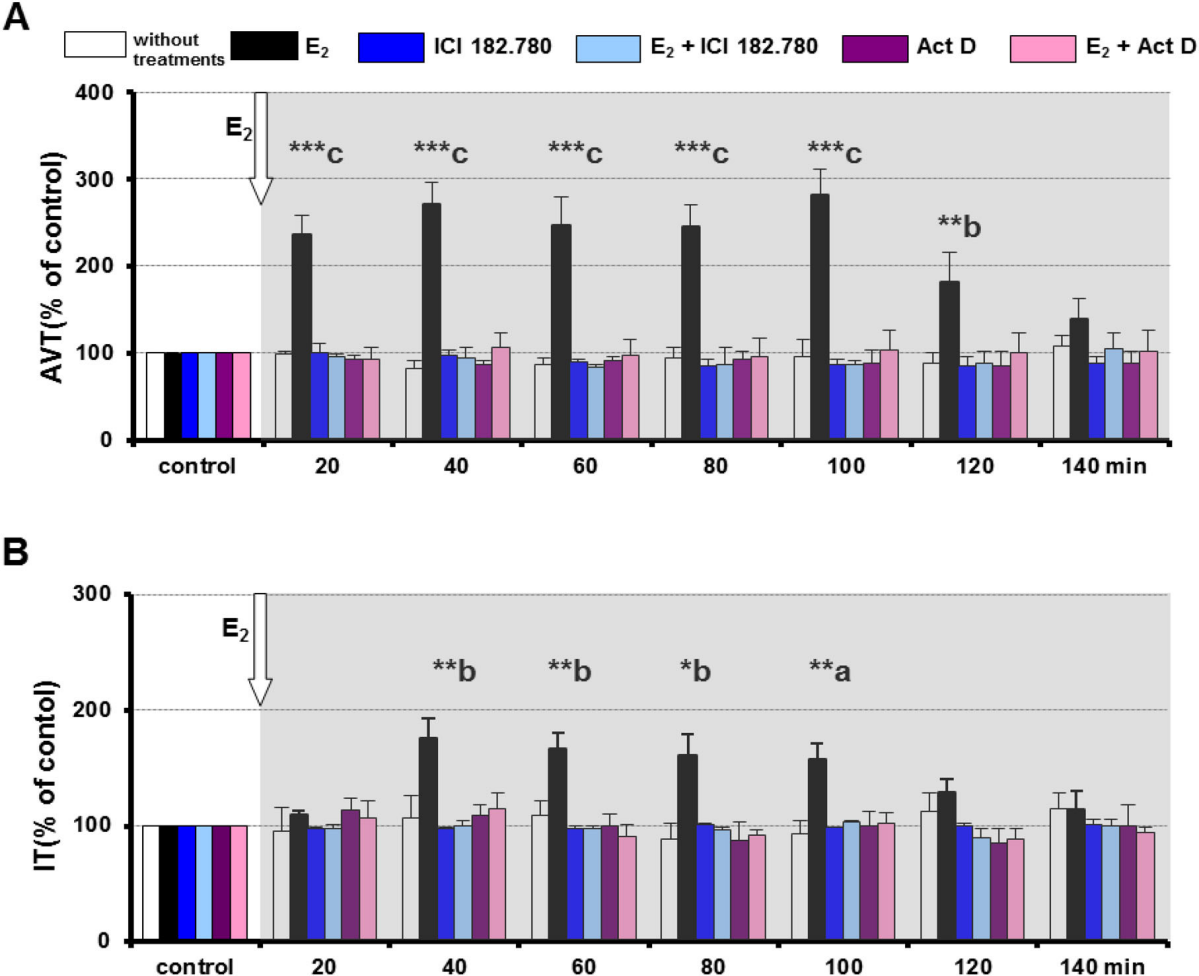
**AVT and IT release after different *in vitro* treatments in female round goby during the spawning-capable phase.** The effect of E2 (3.67×10^−8^ M), Fulvestrant (ICI 82.780; 1×10^−7^ M), Actinomycin D (Act D; 1×10^−7^ M) and E2 in combination with ICI 182.780 or Actinomycin D on AVT (A) and IT (B) release. Values are mean±s.e.m.; *n*=6 for each group. AVT and IT values are expressed as % of the controls. Asterisks above bars indicate differences versus controls: *P<0.05, **P<0.01, ***P<0.001 (two-way ANOVA with Newman–Keuls post hoc test). Letters above bars indicate differences between treatments within a time point of incubation: a, *P*<0.05; b, *P*<0.01; c, *P*<0.001 (one-way ANOVA with Duncan's multiple range test). The grey background indicates the time of E_2_ action.

During the spawning-capable phase, E_2_ (3.67×10^–8^ M) significantly increased AVT release (*P*<0.01; *P*<0.001) compared with the control (Fig. 1A). The AVT response to E_2_ occurred within the first 20 min and persisted for the next 100 min of perfusion. IT release was also stimulated by E_2_ (*P*<0.05; *P*<0.01), but the response appeared after 40 min of perfusion and persisted for the next 60 min (Fig. 1B).

### The influence of Fulvestrant on AVT and IT during the spawning-capable phase

A higher dose of Fulvestrant (1×10^–6^ M) caused a 60% inhibition of AVT and a 50% inhibition of IT release, and therefore the dose was not used in further *in vitro* perfusion experiments (data not shown). A lower dose of Fulvestrant (1×10^–7^ M) did not influence AVT and IT release into perfusion media (Fig. 1A,B); this dose was used further. Fulvestrant (1×10^–7^ M) significantly inhibited the fast response of AVT (*P*<0.01; *P*<0.001) (Fig. 1A) and the slower response of IT (*P*<0.05; *P*<0.01) (Fig. 1B) to E_2_ stimulation (3.67×10^–8^ M) during the spawning-capable phase.

### The influence of Actinomycin D on AVT and IT during the spawning-capable phase

A higher dose of Actinomycin D (1×10^–6^ M) caused a 45% inhibition of AVT release and a 35% inhibition of IT release, and therefore the dose was not used in further *in vitro* experiments (data not shown). A lower dose of Actinomycin D (1×10^–7^ M) did not affect AVT and IT release into perfusion media (Fig. 1A,B), and this dose was used subsequently. Actinomycin D (1×10^–7^ M) significantly inhibited the response of AVT (*P*<0.01; *P*<0.001) (Fig. 1A) to E_2_ (3.67×10^–8^ M) during the spawning-capable phase. The response of IT, induced by E_2_, was also significantly inhibited (*P*<0.05; *P*<0.01) (Fig. 1B) by Actinomycin D during this phase.

### The influence of 17β-oestradiol on AVT and IT during the regressing phase

**Fig. 2.**
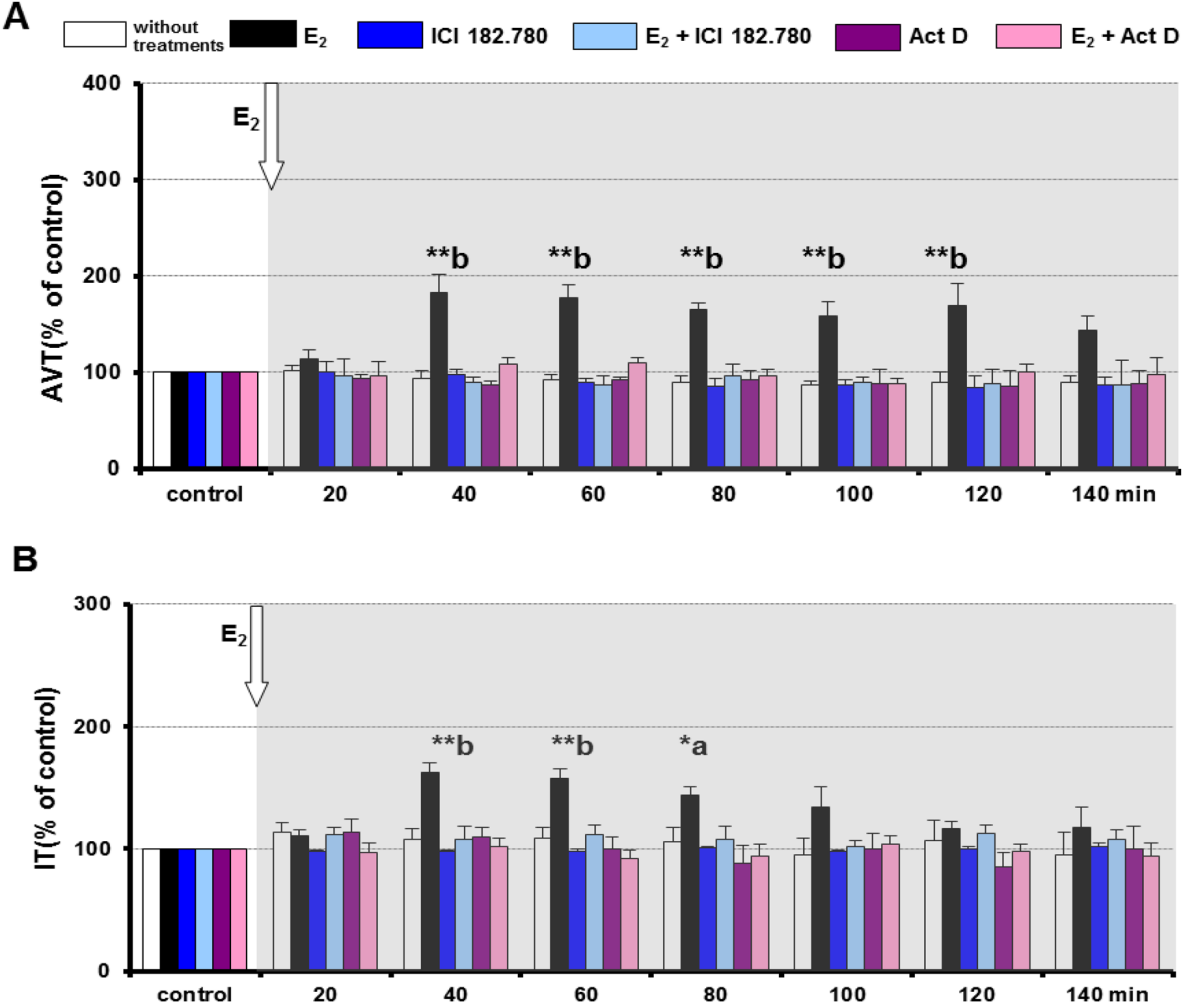
**AVT and IT release following different *in vitro* treatments in female round goby during the regressing phase.** The influence of E2 (1.8×10^−8^ M), Fulvestrant (ICI 182.780; 1×10^−7^ M), Actinomycin D (Act D; 1×10^−7^ M) and E2 in combination with ICI 182.780 or Actinomycin D on AVT (A) and IT (B) release. Values are mean±s.e.m.; *n*=6 for each group. AVT and IT values are expressed as % of the controls. Asterisks above bars indicate differences versus controls: *P<0.05, **P<0.01 (two-way ANOVA with Newman–Keuls post hoc test). Letters above bars indicate differences between treatments within a time point of incubation: a, *P*<0.05; b, *P*<0.01 (one-way ANOVA with Duncan's multiple range test). The grey background indicates the time of E_2_ action.

In the regressing phase, E_2_ (1.8×10^–8^ M) significantly increased AVT (*P*<0.01) and IT release (*P*<0.01; *P*<0.05) compared with the control (Fig. 2A,B). The release of AVT stimulated by E_2_ occurred after 40 min and persisted for the next 80 min of perfusion (Fig. 2A). IT response to E_2_ also appeared after 40 min of perfusion and persisted for the next 60 min (Fig. 2B).

### The influence of Fulvestrant on AVT and IT in the regressing phase

A higher dose of Fulvestrant (1×10^–6^ M) caused results comparable to those obtained during the spawning-capable phase so this dose was not used in further *in vitro* perfusion experiments (data not shown). A lower dose of Fulvestrant (1×10^–7^ M) did not influence AVT and IT release into perfusion media (Fig. 2A,B) and this dose was used subsequently. Fulvestrant (1×10^–7^ M) significantly inhibited the response of both AVT (*P*<0.01) (Fig. 2A) and IT (*P*<0.05; *P*<0.01) (Fig. 2B) to E_2_ stimulation (1.8×10^–8^ M) during the regressing phase.

### The influence of Actinomycin D on AVT and IT during the regressing phase

A higher dose of Actinomycin D (1×10^–6^ M) caused results comparable to those obtained during the spawning phase and so this dose was not used in further *in vitro* experiments (data not shown). A lower dose of Actinomycin D (1×10^–7^ M) did not affect the AVT and IT release (Fig. 2A,B) into perfusion media and this dose was used in subsequent experiments. Actinomycin D (1×10^–7^ M) significantly inhibited the response of both AVT (*P*<0.01) (Fig. 2A) and IT (*P*<0.05; *P*<0.01) (Fig. 2B) induced by E_2_ (1.8×10^–8^ M) during the regressing phase.

### Histological analysis of ovaries

**Fig. 3.**
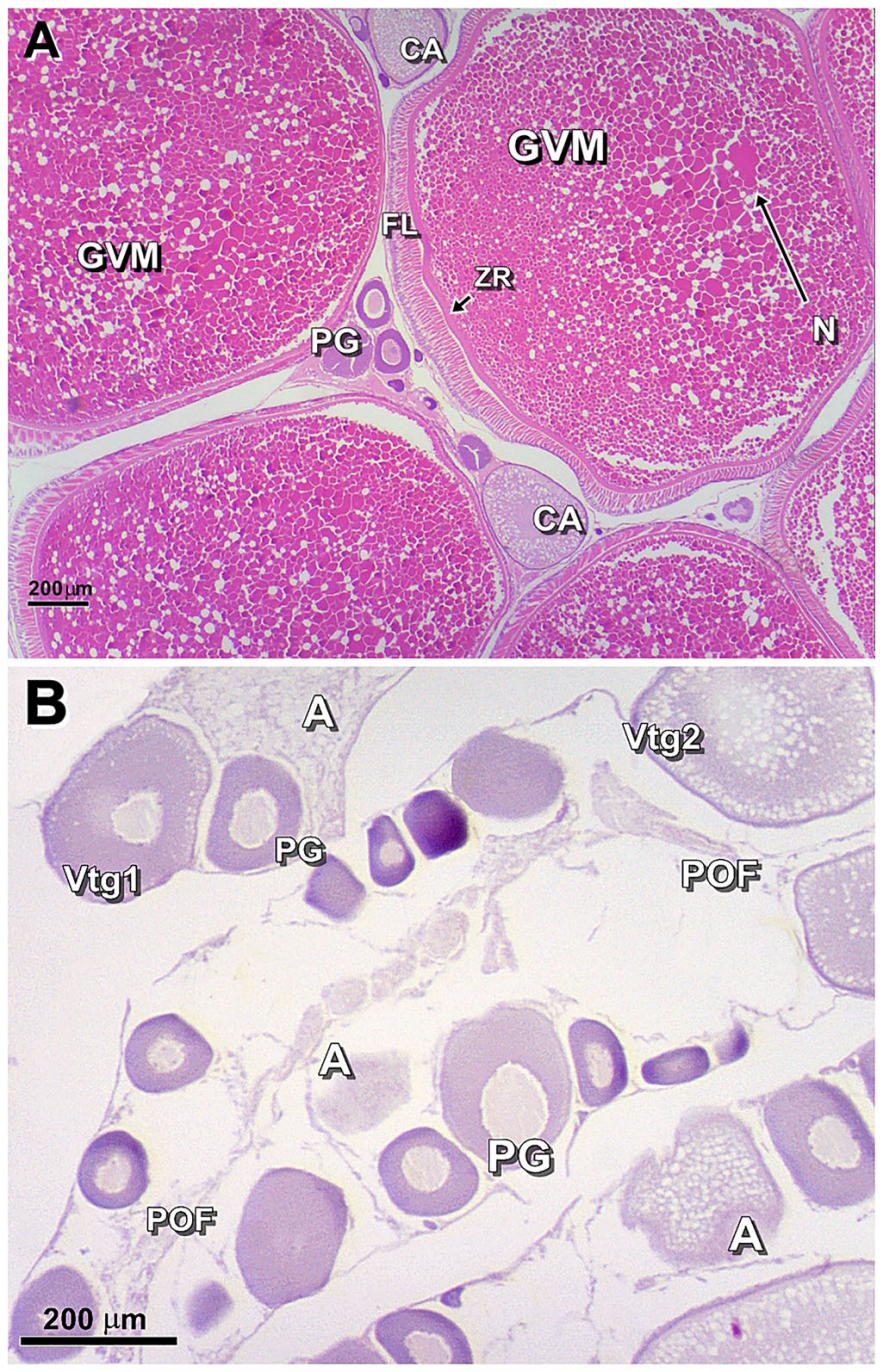
**Oocyte maturation and regression in spawning-capable and regression phases.** Transverse sections of round goby ovaries illustrating oocyte maturation in spawning-capable phase (A) and regression in regressing phase (B). Scale bars: 200 μm. A, atresia; CA, cortical alveoli; FL, follicular layer; GVM, germinal vesicle migration; N, nucleus; PG, primary growth oocyte; POF, post-ovulatory follicular complex; Vtg1, stage 1 vitellogenic oocyte; Vtg2, stage 2 vitellogenic oocyte; ZR, zona radiata.

Ovaries of female round gobies collected during the spawning-capable phase contained follicles at the end of vitellogenic oocyte growth and during oocyte maturation after germinal vesicle migration (GVM) but before germinal vesicle breakdown (Fig. 3A). In contrast, ovaries collected during the regressing phase were regressed and reproductively inactive, containing primary growth oocytes (PG), cortical alveoli (CA), vitellogenic oocytes (Vtg1, Vtg2), atretic oocytes (A) and postovulatory follicle complexes (POFs) (Fig. 3B).

## DISCUSSION

This study presents, for the first time, a presumable mechanism of 17β-oestradiol action on the AVT/IT system in female round goby during the spawning-capable phase and the regressing phase. Our results suggest that changes in oestradiol level related to the phases of the reproductive cycle can affect AVT and IT levels by their ERs. Available information is related only to seasonal changes in gene expression and immunoreactivity of the nonapeptides, which are probably linked with changes in steroid hormones in fish (Ota et al., 1999; Ohya and Hayashi, 2006; Maruska et al., 2007). Based on our studies in stickleback (Gozdowska et al., 2006; Kleszczyńska et al., 2012; Kulczykowska and Kleszczyńska, 2014) and round goby (Sokołowska et al., 2015), we presumed that there is probably a functional link between oestradiol and the AVT/IT system during different phases of the reproductive cycle.

In our experiments, E_2_ was applied at doses mimicking the plasma levels of this hormone in nature during the spawning-capable phase and regressing phase. The doses of E_2_ were established based on their concentrations measured in plasma samples collected during the spawning-capable phase (10±1.2 ng ml^–1^) and the regressing phase (5±1.6 ng ml^–1^). Our findings show that a high level of oestradiol appears at the end of vitellogenic oocyte growth and during oocyte maturation, within the spawning-capable phase in female round goby. In asynchronous spawners, both *in vivo* and *in vitro* studies have shown that the increase in E_2_ production by fully grown ovarian follicles occurs during oocyte maturation (Sakai et al., 1987; Trant and Thomas, 1989). In the study presented here, E_2_ shows a stimulatory effect on AVT during the spawning-capable phase. The secretion of AVT induced by E_2_ increased by ∼140%. It has been noticed that AVT-IR in the preoptic–hypothalamic regions during the peak of the spawning phase was stronger than during the post-spawning phase in female halfspotted goby (*Asterropteryx semipunctata*) and catfish (*Heteropneustes fossilis*) (Maruska et al., 2007; Singh and Joy, 2008). Gozdowska et al. (2006) have demonstrated that the high AVT concentration in female brain in the spawning period was coincident with active reproduction and probably also with high levels of sex hormones. Subsequently, it was indicated that the high AVT level observed before egg laying in female three-spined sticklebacks probably induces the final oocyte maturation (Kulczykowska and Kleszczyńska, 2014). In female catfish, AVT can induce not only the final oocyte maturation but also ovulation through the influence on the production of the maturation-inducing steroid (17α, 20β-dihydroxy-4-pregnen-3-one) (Singh and Joy, 2011). Sokołowska et al. (2015) demonstrated that the high level of brain AVT corresponded with the late, advanced vitellogenesis during the spawning-capable phase in female round goby. Our results showed the fast oestradiol effect (within 20 min) on AVT secretion. The ER antagonists Fulvestrant and Actinomycin D inhibited oestradiol action on AVT secretion during the spawning-capable phase. It is known that in fish, Fulvestrant blocks both genomic and rapid, non-genomic effects of oestradiol via classical nuclear and membrane ERs, respectively (Celius et al., 1999; Loomis and Thomas, 2000; Liu et al., 2011). Therefore, the results presented here indicate that oestradiol most probably stimulates the release of AVT through classical ERs via both genomic and non-genomic pathways during the spawning-capable phase. Moreover, it has been postulated that in fish, as in tetrapods, cross-talk between the non-genomic and genomic pathways of oestradiol signalling may be involved in the control and synchronized maturation and ovulation at the hypothalamic and ovary level (Vasudevan and Pfaff, 2008; Roepke et al., 2009; Cornil and Charlier, 2010; Thomas, 2017). Briefly, rapid effects of E_2_ mediated through mER resulting in the activation of protein kinases can lead to phosphorylation of cAMP-responsive binding protein, which can alter gene transcription through its interaction with the cAMP-responsive element (Cornil et al., 2006, 2012).

During the spawning-capable phase, 17β-oestradiol exerted a stimulatory effect on IT release from brain explants of female round goby. Seasonal fluctuations of IT, which show a higher level during the breeding period, was demonstrated in female three-spined stickleback (Gozdowska et al., 2006). In this species, IT is probably engaged in controlling the final oocyte maturation and/or egg deposition in response to male courtship (Kulczykowska and Kleszczyńska, 2014). However, in female round goby, IT may be related to ovulation rather than late advanced vitellogenesis and oocyte maturation (Sokołowska et al., 2015). Our results showed that, in contrast to AVT, IT secretion stimulated by oestradiol appeared later, after 40 min, and showed a ∼50% increase. In female round goby, oestradiol probably influenced IT secretion by classical nuclear ERs via the genomic pathway only, because Fulvestrant and Actinomycin D inhibited oestradiol action. It is of note that oestrogen may stimulate OT mRNA expression in neuroblastoma cells via the direct genomic pathway (Richard and Zingg, 1990).

The post-spawning gonadal regression phase has received less attention than other phases of the reproductive cycle. The results presented here indicate that the plasma level of E_2_ significantly decreases in female round goby during this phase. The regressing phase is a reproductively inactive period, mainly characterized by the decline of plasma E_2_ levels, a relatively stable and low amount of ERs, and the low expression of ER transcripts in brain and gonads of batch spawners (Burke et al., 1984; Methven et al., 1992; Rinchard et al., 1993; Chen et al., 2011; Nagasawa et al., 2014).

During the regressing phase, E_2_ exerted a stimulatory effect on AVT in female round goby. Nonetheless, the AVT revealed only a ∼70% increase in response to E_2_. In female medaka, halfspotted goby and catfish, AVT-IR in the preoptic–hypothalamic regions was weaker during the post-spawning phase than in the pre-spawning and spawning phases (Ohya and Hayashi, 2006; Maruska et al., 2007; Singh and Joy, 2008). In the non-spawn period, the concentration of AVT in the brain of female three-spined sticklebacks was significantly lower than that during spawning, which is associated with low levels of sex hormones (Gozdowska et al., 2006). Similarly, the low level of brain AVT coincided with the quiescent phase of gametogenesis during the regressing phase in female round gobies (Sokołowska et al., 2015). In rodents, it was demonstrated that during the non-breeding season, the decline in gonadal hormone level is accompanied by decreased AVP expression in several brain regions (Buijs et al., 1986; Hermes et al., 1990; Bittman et al., 1996). In this study, oestrogen-stimulated AVT secretion occurred later, after 40 min of perfusion. Fulvestrant, as well as Actinomycin D, inhibited AVT release affected by E_2_. The results presented here suggest that in the regressing phase, the effect of oestradiol on AVT release is mediated through the classical nuclear ERs via a genomic pathway only.

17β-Oestradiol also had a stimulatory effect on IT in female round goby in the regressing phase. As in the case of AVT, IT displayed a moderate, ∼50%, increase in response to E_2_. It should be noted that IT-IR in the preoptic–hypothalamic regions was weaker in the post-spawning phase than in the pre-spawning phase in female medaka and halfspotted goby (Ohya and Hayashi, 2006; Maruska et al., 2007). In female round goby, the regressing phase was accompanied by a low level of brain IT (Sokołowska et al., 2015). Furthermore, brain IT level declined after egg laying and remained low during winter in female three-spined sticklebacks (Gozdowska et al., 2006; Kulczykowska and Kleszczyńska, 2014). Similar to AVT, IT secretion stimulated by oestradiol appeared after 40 min of perfusion and was inhibited by Fulvestrant and Actinomycin D. Our results suggest that IT release is mediated through classical nuclear ERs via a genomic pathway during this phase of the reproductive cycle.

### Conclusions

Our *in vitro* study presents, for the first time, feasible mechanisms for 17β-oestradiol action on the AVT/IT system in female fish during different phases of the reproductive cycle. During the spawning-capable phase, the effect of E_2_ on AVT release is mediated through classical nuclear and membrane ERs via both genomic and non-genomic pathways, while IT release is mediated through classical nuclear ERs via a genomic pathway only. In the regressing phase, release of both nonapeptides is mediated through classical nuclear ERs via a genomic pathway. The presented mechanism of oestradiol action on the AVT/IT system does not exclude the possibility of action via non-classical mERs, such as ER-X, GPER and Gq-mER.

## MATERIALS AND METHODS

### Experimental fish

Adult female round gobies (*Neogobius melanostomus* Pallas, 1814) (*n*=80) were caught in the Gulf of Gdańsk (Gdynia, Poland) during the spawning-capable phase (April–August) and the regressing phase (September–November). Fish were kept in tanks at the Institute of Oceanology PAS (Sopot, Poland) for 1 week before experimentation. The tanks' water salinity was 8 ppt. Fish were maintained under a lighting regime and temperature according to conditions prevailing in the natural environment. Fish were fed frozen mussels *ad libitum*. Studies were performed on round goby due to their availability and proper size for *in vitro* studies and plasma sample collections and our experience with this species (Kalamarz-Kubiak et al., 2011, 2015; Sokołowska et al., 2015). Before conducting experiments, 20 randomly selected fish were anaesthetized in MS 222 (tricaine methanesulfonate) water solution (50 mg l^–1^) (A5040; Sigma-Aldrich) and blood samples were collected by cardiac puncture. Plasma was separated by centrifuging in heparinized tubes at 3000 ***g*** for 10 min and stored at −70°C prior to E_2_ analysis. Plasma E_2_ concentrations were measured to establish the adequate doses for *in vitro* perfusions that mimic the plasma levels of this hormone in nature during the spawning-capable phase and regressing phase. At the time of sampling, the rest of the fish (*n*=60) were anaesthetized in MS 222 water solution (50 mg l^–1^) (Sigma-Aldrich) and blood samples were collected by cardiac puncture. After transection of the spinal cord, their brains (without pituitaries) were immediately dissected under a stereomicroscope (mikroLAB, Lublin, Poland). Before perfusion, brain explants were washed in Ringer solution supplemented with 10^–6^ mM Bacitracin (11702; Sigma-Aldrich). Blood samples were centrifuged at 3000 ***g*** for 10 min and stored at −70°C prior to E_2_ analysis. In this case, plasma E_2_ concentrations were measured to determine the hormonal status of females. Ovaries collected from freshly euthanized fish were examined morphologically and then the gonads underwent histological analysis. All experiments complied with the EC Directive 2010/63/EU for animal experiments and with the guidelines (19/2012) of the Local Ethics Committee on Animal Experimentation.

The gonadosomic index (GSI) was calculated as (gonad weight/body weight)×100. The mean GSI (%) was 11.14±0.97 for the spawning-capable phase and 1.34±0.19 for the regressing phase. In multiple-spawning fish, GSI seems to be a less reliable indicator of fish maturity than the gonadal histology (Guerriero, 2007; Zeyl et al., 2014). Round goby is a batch spawner; therefore, a histological analysis provides a precise assessment of ovarian maturity and distinguishes between fish in an intermediate spawning stage and those in a post-spawning stage.

### Histology

Ovaries fixed in 4% buffered formalin were dehydrated and embedded in paraffin using standard histological techniques. Embedded tissues were cross-sectioned into 6 μm slices using a Leica RM2245 microtome (Leica Microsystems, Wetzlar, Germany) and stained with Haematoxylin (MHS16; Sigma-Aldrich) and Eosin (861006; Sigma-Aldrich). Slides prepared from each gonad were examined with a Leica HI1210 light microscope (Leica Microsystems). The developmental stage of ovaries was determined according to the terminology developed by Brown-Peterson et al. (2011). This standardized terminology is applicable to all fish regardless of reproductive strategy or gender, including batch-spawning species with asynchronous oocyte development, such as round goby. Batch spawners can exhibit various levels of asynchronous oocyte development and spawn multiple batches of oocytes during the reproductive season (Tomczak and Sapota, 2006; Farrell et al., 2012). The histological analysis was crucial for estimation of the stage of the reproductive cycle and allowed assignment of individuals to groups.

### Perfusion system

All *in vitro* experiments were performed using the perfusion system from MINUCELLS and MINUTISSUE Vertriebs (Bad Abbach, Germany) according to the method developed by Kalamarz-Kubiak et al. (2011). The set used in the experiments consisted of storage medium bottles, a peristaltic pump (ISMATEC, Wertheim, Germany), two gas exchange modules, a gradient perfusion container and plastic vials for the sampling medium after perfusion. The unique structure of the gradient perfusion container facilitates the simultaneous supply of the medium from the top and bottom. In our experiments, two brain explants were perfused simultaneously in a single gradient perfusion container. Brain explants were perfused with Ringer buffer (pH 7.4) prepared according to the composition previously described by Kalamarz-Kubiak et al. (2015). Explants were put on the 20 µm Nylon Net Filter (NY2002500; Merck Millipore, Darmstadt, Germany) placed between the base and tension rings of the tissue carrier (MINUSHEET; diameter of 13 mm) in a gradient culture container. The flowing medium was aerated inside the gas exchange modules by the gas mixture (95% O_2_ and 5% CO_2_) at a pressure of 315.03 mm Hg. Next, a peristaltic pump transported the aerated medium (0.1 ml min^–1^) to the gradient perfusion container and then to the sampling vials. Storage medium bottles, a gradient perfusion container and sampling vials were placed on ice and the media collected after perfusions were stored at −70°C, prior to AVT and IT assay. The perfusions were carried out in a laminar air flow (NUAIRE Biological Safety Cabinet Class II, Plymouth, MN, USA). All chemicals were obtained from Sigma-Aldrich.

### Experimental design

During 240 min of perfusion, 12 fractions of 2 ml each were collected every 20 min. The first 40 min of perfusion was carried out to stabilize incubation conditions; the next 20 min was to establish the base release of AVT and IT (control). A further 180 min was carried out in a medium without any treatments or medium supplemented with different treatments [17β-oestradiol (3301; Merck Millipore), Fulvestrant (I4409; Sigma-Aldrich) and Actinomycin D (1071001; SERVA Electrophoresis, Heidelberg, Germany)]. The brain explants were perfused in medium supplemented with E_2_ at doses mimicking the plasma levels of this hormone in nature. The 17β-oestradiol doses were established based on their concentrations measured in plasma samples collected during the spawning-capable phase (10±1.2 ng ml^–1^) and the regressing phase (5±1.6 ng ml^–1^). The dose of inhibitors was selected based on available data for Fulvestrant (Bouma et al., 2003; Pang and Thomas, 2009; Notch and Mayer, 2011) or our previous experiments for Actinomycin D (Kalamarz-Kubiak et al., 2015). Moreover, before experiments, different doses of inhibitors (1×10^–6^ M; 1×10^–7^ M) were tested (data not shown). Finally, E_2_ at 3.67×10^–8^ M and 1.8×10^–8^ M, separately or in combination with Fulvestrant (1×10^–7^ M) or Actinomycin D (1×10^–7^ M), were used in the experiments. The reactivity of explants was checked according to the method described by Kalamarz-Kubiak et al. (2011). The high K^+^ concentration (56 mM) treatment caused a 480% increase in AVT release and a 350% increase in IT release (data not shown). Our results are consistent with available data (Juszczak, 2002; Orłowska-Majdak et al., 2003).

### 17β-Oestradiol assay

17β-Oestradiol was measured in plasma using a Spectria Estradiol radioimmunoassay (RIA) kit (68633; Orion Diagnostica, Finland) according to the method described previously by Kulczykowska et al. (2015). Concentrations of E_2_ were measured directly from 100 µl plasma without extraction. Iodinated E_2_ with ^125^I was used as a tracer. A standard curve was prepared using six standard dilutions of 50, 150, 500, 1500, 5000 and 15,000 pmol l^–1^. The assay was performed according to the kit manufacturer's instructions with slight modifications. The samples were added to RIA tubes that had been precoated with polyclonal anti-rabbit antiserum. After vortexing for 10 s, the tubes were incubated for 2 h at 37°C, decanted, washed with 1 ml Tween 20 solution and decanted again. The radioactivity in each tube was measured for 1 min using a Wallac Wizard 1470 gamma counter (PerkinElmer Life Science, Shelton, CT, USA). The detection limit of the assay was 37 pmol l^–1^. The intra-assay coefficient of variation was 6.5%. The inter-assay variation was not determined because all samples were measured in the same assay. The mean E_2_ concentrations in the plasma of round gobies during the spawning-capable phase and the regressing phase were 10±1.2 ng ml^–1^ and 5±1.6 ng ml^–1^, respectively.

### AVT and IT analysis

Concentrations of AVT and IT in the media collected after perfusion (2 ml) were determined using HPLC with fluorescence and UV detection, preceded by solid-phase extraction (SPE), according to the modified procedure by Gozdowska et al. (2013). The media after perfusion were acidified with 1 M HCl (258148-M; Sigma-Aldrich) to pH 3–4 and loaded on SPE columns. SPE extraction was carried out on Strata-X (30 mg ml^–1^) columns (8BS100-TAK; Phenomenex, Torrance, CA, USA). The extraction procedure for perfusion media was as follows: samples loaded on conditioned columns [1 ml of 100% methanol (8405; JT Baker Chemicals, Deventer, The Netherlands) then 1 ml H_2_O], then 600 µl H_2_O and 600 µl of 0.1% trifluoroacetic acid (TFA; 302031-M; Sigma-Aldrich) in 5% acetonitrile were passed through the columns to wash away impurities. Hormones were eluted with 2×600 µl of 80% acetonitrile (8149.2500; JT Baker Chemicals). Eluates were evaporated to dryness using TurboVap LVTM (Caliper Life Sciences, PerkinElmer, Waltham, MA, USA). Afterwards, derivatization of peptides was performed using 4-fluoro-7-nitro2,1,3-benzoxadiazole (NBD-F; 47140; Sigma-Aldrich). Dried samples were reconstituted with 40 µl of 0.1% TFA in H_2_O. For the derivatization reaction, 20 µl of the sample, 20 µl of 0.2 M phosphate buffer (pH 9) and 20 µl acetonitrile were mixed and later 3 μl NBD-F (30 mg ml^–1^ acetonitrile) was added. The mixture was heated at 60°C for 3 min, cooled on ice, acidified with 4 μl of 1 M HCl and passed through a HPLC column. Quantitative analyses were performed on the 1200 series Quaternary HPLC system (Agilent Technology, Santa Clara, CA, USA) with a fluorescence detector and a diode array detector. The chromatographic separation of peptides was carried out on ZORBAX Eclipse XDB-C18 (4.6 mm×150 mm, 5 μm) (Agilent Technology). The following optimized chromatographic conditions were used: mobile phase A [0.1% TFA in H_2_O], B [0.1% TFA in acetonitrile: H_2_O (3:1)]; linear gradient system: 45–70% phase B in 12 min. The column temperature was 20°C and the flow rate was 1 ml min^–1^. Fluorescence detection was performed at 470 nm with emission at 530 nm, UV detection at 215 and 340 nm. Recovery of peptides was in the range 79–85% for AVT and IT. The limit of detection was determined to be 0.25 pmol ml^–1^ for AVT and 1.0 pmol ml^–1^ for IT. Intra-day repeatability expressed as relative standard deviation was 6.9–7.9% and 5.3–8.2% for AVT and IT, respectively; inter-day precision was in the range 8.2–9.9 and 5.5–8.5% for AVT and IT, respectively.

### Statistical analysis

Statistical analysis was performed using STATISTICA 7.1 software (StatSoft, http://www.statsoft.pl). Nonapeptide values in media are presented as % of control. A one-way ANOVA followed by Duncan's multiple range test were used to compare the different treatments within each time point of incubation and the same treatment across the time of perfusion. A two-way ANOVA followed by the Newman–Keuls post hoc test were used to compare two different treatments across the time of perfusion. Student's unpaired *t*-test was used to detect differences in plasma E_2_ concentration and a base level of nonapeptides during the spawning-capable phase and the regressing phase (Table 1). *P*<0.05 was considered significant.
